# EVALUATING POPULATION ATTRIBUTABLE FRACTIONS ASSOCIATED WITH ALZHEIMER’S DISEASE RISK FACTORS

**DOI:** 10.1093/geroni/igad104.2749

**Published:** 2023-12-21

**Authors:** Igor Akushevich, Arseniy Yashkin, Svetlana Ukraintseva, Anatoliy Yashin, Julia Kravchenko

**Affiliations:** Duke University, Durham, North Carolina, United States; Duke University, Durham, North Carolina, United States; Duke University, Durham, North Carolina, United States; Social Science Research Institute, Duke University, Durham, North Carolina, United States; Duke University School of Medicine, Durham, North Carolina, United States

## Abstract

Although many risk factors for Alzheimer’s disease (AD) and related dementias (ADRD) have been identified, there is no consensus about their joint role in risk of these disorders. The extent to which joint variation in the effects of behavior, socioeconomic status, comorbidities and genetic variants may affect risk of AD/ADRD is underexplored. In this study, we used Health and Retirement Study (HRS) data linked to Medicare diagnoses and genetic data to evaluate and compare the effects of three groups of AD/ADRD risk factors: i) Medicare diagnoses for AD risk-related diseases, ii) survey-based self-reported health, behavior, physical function, education, and socioeconomic status, and iii) individual SNPs and polygenic risk scores incorporating genetic risk factors for AD/ADRD. We applied univariable and multivariable Cox models for estimating AD and ADRD risks, identified most powerful predictors, evaluated their population attributable fractions, and constructed predictive multivariable models for AD/ADRD risks. The results showed that Medicare-based diagnoses are superior in terms of predictive power of AD/ADRD risk than their self-reported counterparts. Cerebrovascular and heart diseases, depression, and brain injury had the largest effect on AD/ADRD risk. Depression and arterial hypertension were the largest contributors to AD risk in population. These two factors and APOE4 proxy SNP rs769449, together explained approximately half of the population attributable fraction of AD risk. The effect of PRS on AD/ADRD was significant for genes involved in inflammatory pathway. Our study demonstrated that AD/ADRD risk is multifactorial in nature and its variation is tied to multiple demographic, socioeconomic and health-related risk factors.

